# GBS-based single dosage markers for linkage and QTL mapping allow gene mining for yield-related traits in sugarcane

**DOI:** 10.1186/s12864-016-3383-x

**Published:** 2017-01-11

**Authors:** Thiago Willian Almeida Balsalobre, Guilherme da Silva Pereira, Gabriel Rodrigues Alves Margarido, Rodrigo Gazaffi, Fernanda Zatti Barreto, Carina Oliveira Anoni, Cláudio Benício Cardoso-Silva, Estela Araújo Costa, Melina Cristina Mancini, Hermann Paulo Hoffmann, Anete Pereira de Souza, Antonio Augusto Franco Garcia, Monalisa Sampaio Carneiro

**Affiliations:** 1 Departamento de Biotecnologia e Produção Vegetal e Animal, Centro de Ciências Agrárias, Universidade Federal de São Carlos, Rodovia Anhanguera, Km 174, Araras, CEP 13600-970 São Paulo Brazil; 2 Departamento de Biologia Vegetal, Instituto de Biologia, Universidade Estadual de Campinas, Avenida Monteiro Lobato 255, Campinas, CEP 13083-862 São Paulo Brazil; 3 Centro de Biologia Molecular e Engenharia Genética, Universidade Estadual de Campinas, Avenida Candido Rondon 400, Campinas, CEP 13083-875 São Paulo Brazil; 4 Departamento de Genética, Escola Superior de Agricultura Luiz de Queiroz, Universidade de São Paulo, Avenida Pádua Dias 11, Piracicaba, CEP 13418-900 São Paulo Brazil

**Keywords:** *Saccharum* spp, Polyploidy, SNPs, Molecular markers, Allelic dosage, Quantitative traits

## Abstract

**Background:**

Sugarcane (*Saccharum* spp.) is predominantly an autopolyploid plant with a variable ploidy level, frequent aneuploidy and a large genome that hampers investigation of its organization. Genetic architecture studies are important for identifying genomic regions associated with traits of interest. However, due to the genetic complexity of sugarcane, the practical applications of genomic tools have been notably delayed in this crop, in contrast to other crops that have already advanced to marker-assisted selection (MAS) and genomic selection. High-throughput next-generation sequencing (NGS) technologies have opened new opportunities for discovering molecular markers, especially single nucleotide polymorphisms (SNPs) and insertion-deletion (indels), at the genome-wide level. The objectives of this study were to (i) establish a pipeline for identifying variants from genotyping-by-sequencing (GBS) data in sugarcane, (ii) construct an integrated genetic map with GBS-based markers plus target region amplification polymorphisms and microsatellites, (iii) detect QTLs related to yield component traits, and (iv) perform annotation of the sequences that originated the associated markers with mapped QTLs to search putative candidate genes.

**Results:**

We used four pseudo-references to align the GBS reads. Depending on the reference, from 3,433 to 15,906 high-quality markers were discovered, and half of them segregated as single-dose markers (SDMs) on average. In addition to 7,049 non-redundant SDMs from GBS, 629 gel-based markers were used in a subsequent linkage analysis. Of 7,678 SDMs, 993 were mapped. These markers were distributed throughout 223 linkage groups, which were clustered in 18 homo(eo)logous groups (HGs), with a cumulative map length of 3,682.04 cM and an average marker density of 3.70 cM. We performed QTL mapping of four traits and found seven QTLs. Our results suggest the presence of a stable QTL across locations. Furthermore, QTLs to soluble solid content (BRIX) and fiber content (FIB) traits had markers linked to putative candidate genes.

**Conclusions:**

This study is the first to report the use of GBS for large-scale variant discovery and genotyping of a mapping population in sugarcane, providing several insights regarding the use of NGS data in a polyploid, non-model species. The use of GBS generated a large number of markers and still enabled ploidy and allelic dosage estimation. Moreover, we were able to identify seven QTLs, two of which had great potential for validation and future use for molecular breeding in sugarcane.

**Electronic supplementary material:**

The online version of this article (doi:10.1186/s12864-016-3383-x) contains supplementary material, which is available to authorized users.

## Background

Sugarcane (*Saccharum* spp.) has a complex genome because of its variable ploidy level, frequent aneuploidy and large genome size of approximately 10 Gb [1–5]. This crop is a member of the Poaceae family and the Andropogoneae tribe, which includes maize and sorghum [6, 7]. Modern sugarcane cultivars are the result of interspecific crosses between the domesticated species *Saccharum officinarum* L. (2n = 80) and the wild species *S. spontaneum* L. (2n = 40–120), followed by several backcrosses with *S. officinarum* [6, 8]. These cultivars have chromosome numbers ranging from 100 to 130, are vegetatively propagated, and result from the selection of populations derived from outcrossing heterozygous parents [1, 9]. Sugarcane has a very high photosynthetic efficiency and is a crop with major economic importance in many tropical and subtropical countries primarily because of its use in the production of sugar and bioethanol [10–12].

Polyploidy, an important driver of plant evolution in natural populations, has played a crucial role in the domestication of crops such as wheat, sugarcane, cotton and potato [13–16]. Sugarcane is predominantly an autopolyploid plant, and the understanding of its genome organization is limited [4, 7]. One possible way to increase knowledge of the genome organization of this species is by using genetic maps. High-resolution genetic linkage mapping may be used for quantitative trait loci (QTL) studies in mapping poulations and also such as a first step toward potential marker-assisted selection (MAS) in plants [17–22]. Several genetic linkage maps of sugarcane have been generated since a methodology based on single-dose markers (SDMs) was proposed by Wu et al. [23]. SDMs that segregate 1:1 and 3:1 in full-sib progenies (F_1_ populations) [24] or 3:1 in populations created by selfing an individual are commonly used for constructing genetic maps in sugarcane [4, 25–35]. An integrated map of sugarcane with different types of molecular markers, such as microsatellites or single sequence repeats (SSRs) and target region amplification polymorphism (TRAP), extended the characterization of polymorphic variation throughout the entire genome [36–38]. However, in outcrossing heterozygous species such as sugarcane, for each segregating loci, different numbers of segregating alleles can exist, and a relative large number of markers is required to guarantee reasonable coverage of its genome [37, 39, 40].

Currently, high-throughput next-generation sequencing (NGS) technologies have provided new opportunities for discovering molecular markers, especially single nucleotide polymorphisms (SNPs), at the genome-wide level [41–43]. Some of these techniques, *e.g.*, restriction-site associated DNA sequencing (RAD-seq) [44] and genotyping-by-sequencing (GBS) [42, 45, 46], employ a reduced genome representation that is achieved through restriction enzyme digestion, which could be particularly helpful for a complex genome such as that of a polyploid [47]. Moreover, these strategies can be used to species without reference sequence [48]. The GBS protocol has been widely used in a range of genetic studies in several species such as apple, barley, lettuce, switchgrass, maize, rice, wheat, and soybean [42, 45, 46, 49–55]. SNP datasets generated from GBS can be analyzed to detect associations between genotypes and phenotypes, perform diversity analyses, and construct genetic maps, among other applications [39, 56–58].

QTL mapping in sugarcane is a promising tool for characterizing the genetic architecture of several yield component traits of interest, such as sucrose yield, cane yield, stalk diameter, stalk height, stalk number, and stalk weight, as well as resistance to diseases, pests and abiotic stresses [10, 59–62]. Sugarcane is a semi-perennial crop with repeated measures data obtained for several harvests and locations, and QTL mapping studies are usually performed in two steps. First, adjusted phenotypic means are obtained; second, these means are searched for associations with molecular markers and/or along genetic maps [31, 32, 63]. Gazaffi et al. [62] proposed a method that considers an integrated genetic map in which QTL mapping is performed based on the advantages of the composite interval mapping (CIM) approach [64]. Briefly, a mapping model with three genetic effects is considered for genome scanning [62]. It is assumed that a QTL may also segregate in different patterns in progeny as a function of its genetic effects and of the linkage phase between markers and QTL alleles.

This study is the first to report on the development and application of GBS for mapping studies in sugarcane. Our objectives were to (i) establish a pipeline for identifying SNPs and insertion-deletion (indels) from GBS data in a sugarcane F_1_ population, (ii) construct the first GBS-based integrated genetic map with additional SSR and TRAP markers in this bi-parental mapping population, (iii) identify QTLs related to yield component traits based on the integrated genetic map, and (iv) perform annotation of the sequences that originated the associated markers with mapped QTLs to search putative candidate genes that may be involved in yield traits in sugarcane. We discuss these results in the context of where GBS is likely to be most useful in sugarcane crop development.

## Methods

### Mapping population and DNA extraction

The mapping population consisted of 151 full sibs derived from a commercial cross between the SP80-3280 (female parent) and RB835486 (male parent) sugarcane cultivars. The parents are broadly cultivated throughout Brazil because of their high biomass and sugar yields. SP80-3280 (SP71-1088 × H57-5028) was one of the cultivars with transcriptome sequencing performed previously by SUCEST [65] and RNA-seq [66] projects; its genome is currently being completely sequenced by the Brazilian initiative [67]. This cultivar is resistant to brown rust (*Puccinia melanocephala*), whereas RB835486 (L60-14 × ?) is susceptible to fungal disease. The parents have been used in studies of evolutionary relations in putative tandem gene duplication [68] and retrotransposon-based insertion polymorphisms [69]. Total genomic DNA samples from parents and progeny were extracted from the 1+ internode (leaf primordia) as proposed by Al-Janabi et al. [70], with modifications.

### GBS-based markers

GBS was performed by the Institute for Genomic Diversity (Cornell University, Ithaca, NY, USA) according to the protocol described in detail by Elshire et al. [45]. Samples from both parents of the population were replicated three times for sequencing. Each individual within a library was part of a 96-plex reaction (including one blank sample each). To provide a higher sequence depth, libraries were obtained by digestion with *Pst*I, a partially methyl-sensitive six-base-pair site restriction enzyme. Additionally, the 96-plex libraries were run in two distinct lanes each on a HiSeq™ 2000 platform (Illumina® Inc., San Diego, CA, USA).

To discover polymorphisms, we initially used the Tassel-GBS pipeline [71], which was implemented in Tassel software (v. 4.3.8). Because this pipeline requires a reference genome and because the complete sugarcane genome sequencing is in progress [67], we proposed the use of four alternative pseudo-references: (i) a methyl-filtered sugarcane genome (~674 Mb arranged in 1,109,444 scaffolds) [72], (ii) the *Sorghum bicolor* genome (v. 2.1; ~726 Mb arranged in 10 chromosomes and 1,600 unassembled scaffolds) [73], (iii) an RNA-seq sugarcane transcriptome (~780 Mb arranged in 119,768 transcripts) [66], and (iv) sequences from the SUCEST project (~152 Mb of a total of 237,954 sequences) [65]. The Bowtie2 (v. 2.2.1) algorithm was used to map the 64-bp-long tags against each reference with default parameters and the very sensitive-local argument. The exact reference and alternative sequence depths (read counts) were recorded in variant call format (VCF) files. To perform this task, we modified the GBS-Tassel pipeline to record a maximum value of 32,767 counts for each allele.

#### Allelic dosage estimation and marker curation

The GBS technique generated allele-specific read count data in the form *D* = {(*x*_1_, *y*_1_), (*x*_2_, *y*_2_), …, (*x*_*n*_, *y*_*n*_)} for each biallelic locus from individuals *i* = 1, 2, …, *n*. Data *D* from each locus were analyzed in SuperMASSA software [74]. As a prior quality control, markers with more than 25% missing data were filtered out. We also excluded GBS loci data with fewer than 50 read counts for the reference allele on average. In addition, individual data points with the radial coordinate ${r}_i=\sqrt{x_i^2+{y}_i^2}$ smaller than (0.10) × *max*(*r*_1_, *r*_2_, …, *r*_*n*_) were removed.

All even-numbered ploidy levels ranging from 2 to 20 were tested [39]. The ploidy that returned the highest likelihood was selected after fitting a subjacent F_1_ segregation model into SuperMASSA. The replicated parental data provided additional constraints during estimation. Following the recommendation reported in Serang et al. [74] to find the maximum *a posteriori* (MAP) solution for the estimates, the SuperMASSA naive posterior report threshold was set to zero. Afterward, the values of individual posterior probability given the selected ploidy (6 through 14) were also calculated; these values indicated the maximum threshold that would allow individual assignment to a certain dosage cluster. Only ploidies ranging from 6 to 14 were selected because they were more likely to appear in the sugarcane genome and exhibited a greater number of SDMs [39].

For posterior quality control, we only selected SDMs for which the median of all individual posterior probabilities was higher than 0.80. The SuperMASSA dosage outputs were recoded for mapping purposes in R software by substituting the respective reference and alternative codominant alleles for *a* and *b*. Redundant loci within each reference and between references were inspected and excluded based on the recoded genotype calls. Here, only non-redundant loci were used for linkage mapping analysis. A circular plot was used to summarize the duplicate loci within and between references using the R circlize package [75].

### Gel-based SSR and TRAP markers

A total of 120 SSR markers were genotyped in the 151 full-sib progeny and in the two parents. SSR markers were derived from both ESTs and genomic sequences. There were 98 EST-SSRs named SCA [76, 77], SCB [76], SCC [76, 78] and IISR [79], and 16 genomic SSRs named SMC [80] and CIR [81]. In addition, there were six SSR markers (named SB, Xtxp, CNL and SvPEPCAA [82–84]) from a genic sorghum library. PCRs were performed in a final volume of 20 μL as described by Oliveira et al. [76].

For TRAP markers four fixed and three arbitrary primers (named ARB1, ARB2 and ARB3) were used. The arbitrary primers were adapted of Li and Quiros [85]. Two fixed primers were designed from *sucrose phosphate synthase* (SuPS) [86, 87], and one primer each was designed from *caffeic acid 3-O-methyltransferase* (COMT) and *cinnamoyl-CoA reductase* (CCR) [88] gene sequences. PCRs were performed in a final volume of 20 μL [89].

Amplicons of SSR and TRAP markers were denatured at 90 °C for 3 min in an equal volume of loading buffer (formamide containing 0.8 mM EDTA and traces of bromophenol blue and xylene cyanol), snap-cooled on ice, and electrophoresed in 6% denaturing polyacrylamide gels in 1X TBE buffer. The samples were loaded on a dual vertical electrophoresis system (CBS Scientific) and were run at 75 W for 1 to 3 h depending on the fragment sizes to be separated. A 10-bp ladder was used as a standard size. The bands were visualized by silver staining according to Creste et al. [90].

### Linkage map construction and homo(eo)logous group assignment

Linkage mapping analysis was performed over non-duplicated SDMs using the Onemap (v. 2.0-4) R package [91]. This analysis allowed simultaneous estimation of linkage and linkage phases between markers [92] and marker ordering using multipoint likelihood through hidden Markov models [93, 94] from a mixed set of different marker segregation patterns. The markers were coded according to the notation proposed by Wu et al. [92]. In brief, the codominant alleles were coded as *a* and *b,* while the null alleles were coded as *o* and treated as recessive alleles. GBS-based codominant markers were used to assess segregation with the following three cross types: ‘B3.7’ (*ab* × *ab*), ‘D1.10’ (*ab* × *aa*) and ‘D2.15’ (*aa* × *ab*). In addition, SSR and TRAP gel-based dominant markers were used to assess three more cross types that are traditionally used in integrated sugarcane maps: ‘C.8’ (*ao* × *ao*), ‘D1.13’ (*ao* × *oo*) and ‘D2.18’ (*oo* × *ao*). ‘D1’ and ‘D2’ stand for crosses in which the marker locus is heterozygous (and hence informative) only to SP80-3280 or to RB835486, respectively; they are both expected to segregate in a 1:1 ratio. ‘B3’ and ‘C’ stand for crosses in which the marker locus is heterozygous and symmetric in both parents; the former is expected to segregate in a 1:2:1 ratio, whereas the latter will segregate in a 3:1 ratio. Because SuperMASSA is able to predict parental genotypes using the population data even when parental data are missing, all B3-type markers could be recovered. However, D1- and D2-type markers were only recovered when read counts for at least one parental were available; with no parental data, these markers were discarded.

For gel-based markers, chi-square tests were conducted in R software according to the expected segregation ratios inferred through parental genotypes, and then *p*-values were corrected using false discovery rate control for non-dependent tests as implemented in the ‘p.adjusted’ R function. For GBS-based markers, segregation had already been considered during dosage estimation in SuperMASSA software according to the F_1_ model [74].

To obtain the genetic map, we first performed a two-point test to identify linkage groups (LGs). Any pairwise markers that showed a *LOD* Score > 9.0 and a recombination fraction < 0.10 were considered linked. Afterward, we applied ordering algorithms to each group. For the groups with less than six markers, the best order was obtained by performing an exhaustive search with the ‘compare’ function. For those groups with more than six markers, the ‘order.seq’ command was used, *i.e.*, an initial set of the five most informative markers (preferentially B3- and C-type markers) was sampled for an exhaustive search. The best order was used as a frame for the consecutive inclusion of new markers. Once these groups were obtained, we used the ‘try.seq’ function to verify markers that were considered unlinked according to the initial procedure, and it was possible to integrate the pre-ordered groups. In this step, the following other markers were also tested: (i) markers at the ends of the LGs more than 20 centiMorgans (cM) far from the closest marker; and (ii) markers belonging to very small LGs (with sizes less than 1 cM or containing only two loci). As a final step, the LGs with more than five markers were refined using the ‘ripple’ algorithm within a sliding window of five markers. The ordered group heatmap plots were inspected visually, and manual correction was performed when needed throughout the map building process. The LGs were drawn in MapChart software [95]. The homo(eo)logous groups (HGs) were defined according to the sugarcane reference scaffolds shared by the GBS-based markers. Gel-based markers were also checked because they can produce different alleles that share the same primer pair.

### Phenotypic data

The mapping population was planted in 2010 at two locations (Araras, located at 22°21′25″ S, 47°23′03″ W, and Ipaussu, located at 23°08′44″ S, 49°23′23″ W; both in the State of Sao Paulo, Brazil) and evaluated during three harvest years for several yield component traits, including sucrose content of cane (POL%C, in %), soluble solid content (BRIX, in °Brix), stalk diameter (SD, in mm) and fiber (FIB, in %). At each location, the experimental design consisted of an augmented randomized incomplete block design, which was fully replicated three times. For each trait, a multiple-harvest-location trial was considered under a mixed linear model approach for each yield component [10].

### QTL mapping

The joint adjusted phenotypic means by location for each trait were used for QTL mapping. The QTL mapping methodology applied in this work was presented by Gazaffi et al. [62], and it expands the CIM method [64] to full-sib families. In brief, the model has three genetic effects, with two for additive effects (one for each parent) and one dominance effect (intra-loci interaction). To infer the conditional probabilities of QTL genotypes, multipoint probabilities were obtained using hidden Markov models at each 1 cM from the genetic map.

The mapping strategy was based on three steps. First, an interval mapping (IM) [96] search was carried out in order to select marker cofactors. The peaks with a *LOD* Score greater than 2 were sampled for inclusion in the QTL detection procedure. If the peak was not coincident with a marker, the closest one was considered as cofactor. Second, the QTL search was performed along the genome and considered the cofactors located outside the linkage group under analysis. To declare a QTL, the threshold for each search was obtained from 1,000 permutations with a significance level of 0.95 [97]. Finally, the peaks above the permutation threshold were fully characterized, *i.e.*, the significance of each genetic effect was tested along with the linkage phase between markers and QTLs and the QTL segregation pattern. The proportions of phenotypic variance (*R* ^2^) as explained by each detected QTL were obtained for all the effects simultaneously. All the analyses were performed in R software [98].

### Sequence annotation

Functional annotation of the regions of adjacent markers from the mapped QTLs was performed using sequence information from the scaffolds of the methyl-filtered sugarcane genome, sugarcane transcriptome from RNA-seq assembly, sequences from the SUCEST project and sequences with 400 nucleotides in length at both sides of the SNP/indel position for mapped markers from the sorghum genome. These scaffolds and sequences were annotated using Blast2GO software version 3.1 [99] on the non-redundant NCBI database with E values ≤ 1 × 10^−3^, and the Phytozome website [100] was used to align the data against the *Viridiplantae* protein databases.

## Results

### GBS-based marker polymorphism discovery

Short-read sequences were obtained from the mapping population and triple-replicated parents after double sequencing the 96-plex *Pst*I libraries. Of the 330 million good barcoded reads, more than 3.1 million resulting tags were obtained for alignment against four different pseudo-references. Three of four pseudo-references originated from sugarcane DNA or RNA libraries. The methyl-filtered sugarcane genome resulted in the highest alignment rate, with 87.94% (2,729,457) aligned tags. Regarding RNA-based references, 38.53% (1,195,723) and 23.89% (741,537) tags were aligned with the RNA-seq transcriptome and SUCEST project sequences, respectively; however, their rates of non-unique alignment differed greatly (Additional file 1: Table S1). Finally, the reference for the close-relative genome of sorghum had 42.29% (1,312,661) aligned tags.

From 39,058 to 151,755 biallelic variants were identified, depending on the reference. Furthermore, for all the references, SNPs were identified more often than indels, at a 2.6:1 ratio on average (Table 1). With respect to SNPs, transitions (purine-purine or pyrimidine-pyrimidine interchanges) were identified 1.4 times more often than transversions (purine-pyrimidine interchanges). Approximately 12% of the markers with more than 25% missing data were filtered out per reference (Table 1) because current missing data imputation methods are not able to handle the complexity of the sugarcane genome. Low-coverage or ambiguous loci were also broadly present. At this stage of analysis, ~64% underrepresented loci were also excluded by considering a minimum of 50 read counts on average for the reference alleles. Although a large number of loci were removed, from 8,885 to 38,378 high-coverage loci, missing-filtered polymorphic loci were subjected to SuperMASSA quantitative genotyping analyses. The remaining redundancy was investigated only after these analyses.

**Table 1:** Number of markers generated after GBS-Tassel pipeline analyses to map the GBS sugarcane population data

GBS-Tassel pipeline pseudo-references	SNPs	Indels	Total	Excluded data		Filtered polymorphic sites
				Missing data^a^	Low coverage loci^b^	
Methyl-filtered sugarcane genome	110,261	41,494	151,755	16,815 (11.1%)	96,562 (63.6%)	38,378 (25.3%)
*Sorghum bicolor* genome (v. 2.1)	84,757	35,447	120,204	13,773 (11.5%)	78,914 (65.6%)	27,517 (22.9%)
RNA-seq sugarcane transcriptome	73,275	26,778	100,053	11,809 (11.8%)	63,658 (63.6%)	24,586 (24.6%)
SUCEST project sequences	29,238	9,820	39,058	4,878 (12.5%)	25,295 (64.8%)	8,885 (22.7%)

Notes

^a^ More than 25% of the population is missing data

^b^ Less than 50 reads on average for the reference alleles

### Ploidy and allelic dosage estimation

**Fig. 1:**
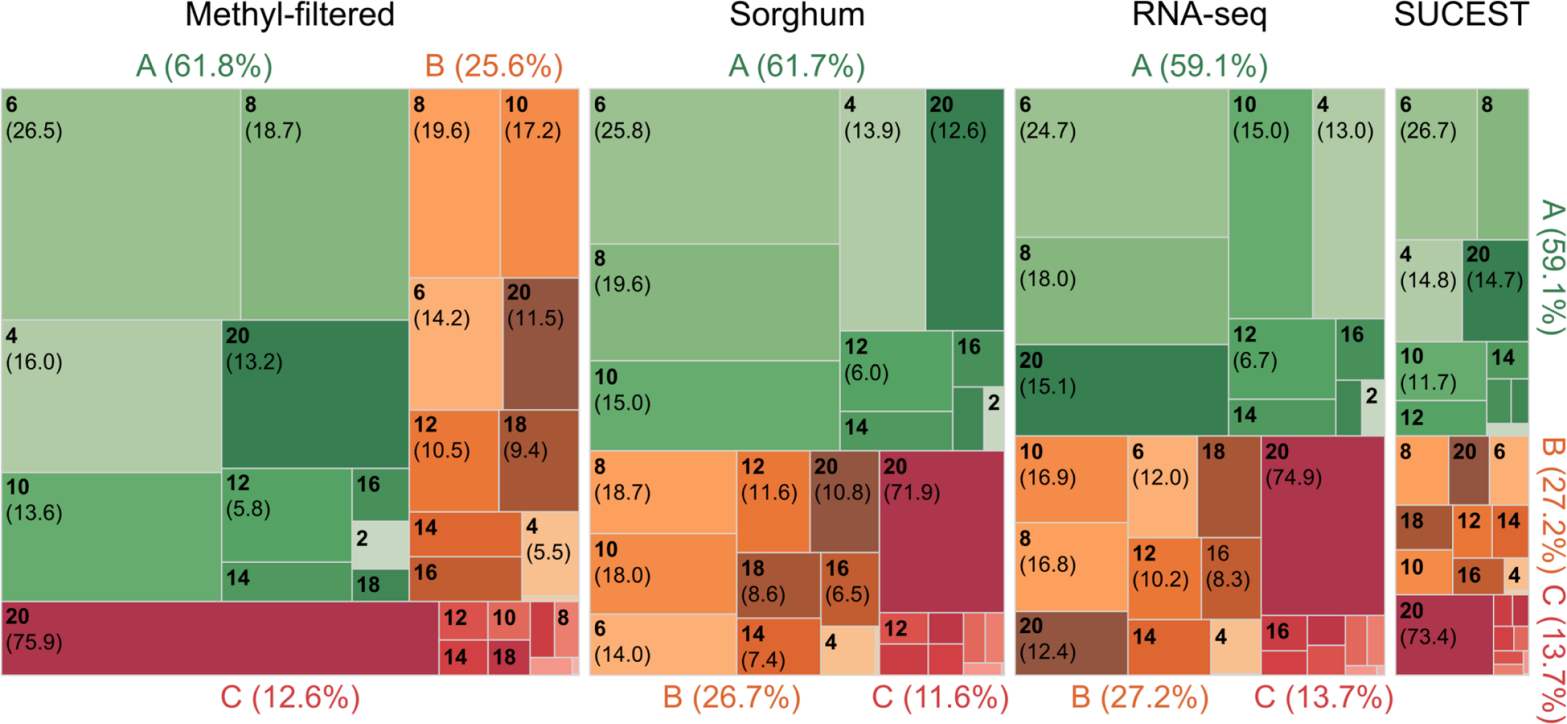
Mosaic plot showing the ploidy levels that produced the highest posterior probabilities for the mapping of GBS sugarcane population data considering the following four pseudo-references: the methyl-filtered sugarcane genome, *Sorghum bicolor* genome, RNA-seq sugarcane transcriptome and sequences from the SUCEST project. The areas of the rectangles are proportional to the number of loci that have the same ploidy level, as indicated within each rectangle in parentheses. According to the posterior probabilities calculated for each even-numbered ploidy level within a range from 2 to 20, each locus was classified into one category using the following *ad hoc* criteria: Category A (*green*), when the highest posterior probability was greater than or equal to 0.80; Category B (*yellow*), when no single value of the posterior probability was higher than 0.80 but the sum of the two highest ones was greater than or equal to 0.80; and Category C (*red*), which included all other cases. In parentheses: the number of loci as a percentage within the given ploidy level and category

Ploidy levels ranging from 2 to 20 were evaluated with SuperMASSA software. Once the more likely ploidy was acknowledged, the software provided the individual posterior probability for each individual that was allocated in one of the expected dosage clusters. For the ploidy levels considered in these analyses, the number of loci varied within each ploidy class (Fig. 1), and an average of 10.7% loci were classified as having ploidies of 2 or 4, 60.3% as ploidies 6 through 14, and 29.0% as ploidies 16 through 20. Here, we used the same *ad hoc* criteria to classify each locus into one quality category based on their posterior probabilities for each ploidy; categories A and B included loci with either the highest or the sum of the two highest posterior probabilities that were greater than or equal to 0.80, respectively, and category C included all other cases [39]. Categories A, B and C represented 60.4%, 26.6% and 12.9% of the loci on average for all the ploidies, respectively (Fig. 1).

For the linkage map construction, we selected the loci that were classified into category A and the ploidies ranging from 6 to 14, which represented 40.7% of loci on average from the total input in SuperMASSA or from 3,433 to 15,906 markers depending on the reference (Table 2). In addition, we characterized these remaining good-quality loci according to dosage. SDMs and multi-dose markers (MDMs) were equally represented by GBS, with approximately 50% on average for each one. As a final quality control analysis of the GBS data, we selected the loci with the median of all individual posterior probabilities greater than 0.80. This *ad hoc* criterion aimed to ensure that the loci used in genetic mapping had at least 50% of the individuals with a high probability (superior to 0.80) of being in their given clusters for the selected ploidy. Depending on the number of clusters, the SDMs were classified as segregating in a 1:2:1 ratio (three clusters) or in a 1:1 ratio (two clusters). On average, the percentages that represented each segregation class were 16.4% and 83.6%, respectively (Table 2).

**Table 2:** Selected loci with good quality (category A) and ploidy (6 through 14) were classified as single-dose markers (SDMs) or multi-dose markers (MDMs). High-quality SDMs (median of all individual *a posteriori* probabilities > 0.80) were also characterized according to their segregation pattern in the sugarcane mapping population

Reference	Total	Dosage		High-quality SDM	Segregation pattern	
		MDM	SDM		1:2:1	1:1
Methyl-filtered sugarcane genome	15,906	7,014 (44.1%)	8,892 (55.9%)	5,266	912 (17.3%)	4,354 (82.7%)
*Sorghum bicolor* genome (v. 2.1)	11,789	5,784 (49.1%)	6,005 (50.9%)	3,433	605 (17.6%)	2,828 (82.4%)
RNA-seq sugarcane transcriptome	9,808	4,959 (50.6%)	4,849 (49.4%)	2,869	469 (16.4%)	2,400 (83.6%)
SUCEST project sequences	3,433	1,736 (50.6%)	1,697 (49.4%)	983	141 (14.3%)	842 (85.7%)

**Fig. 2:**
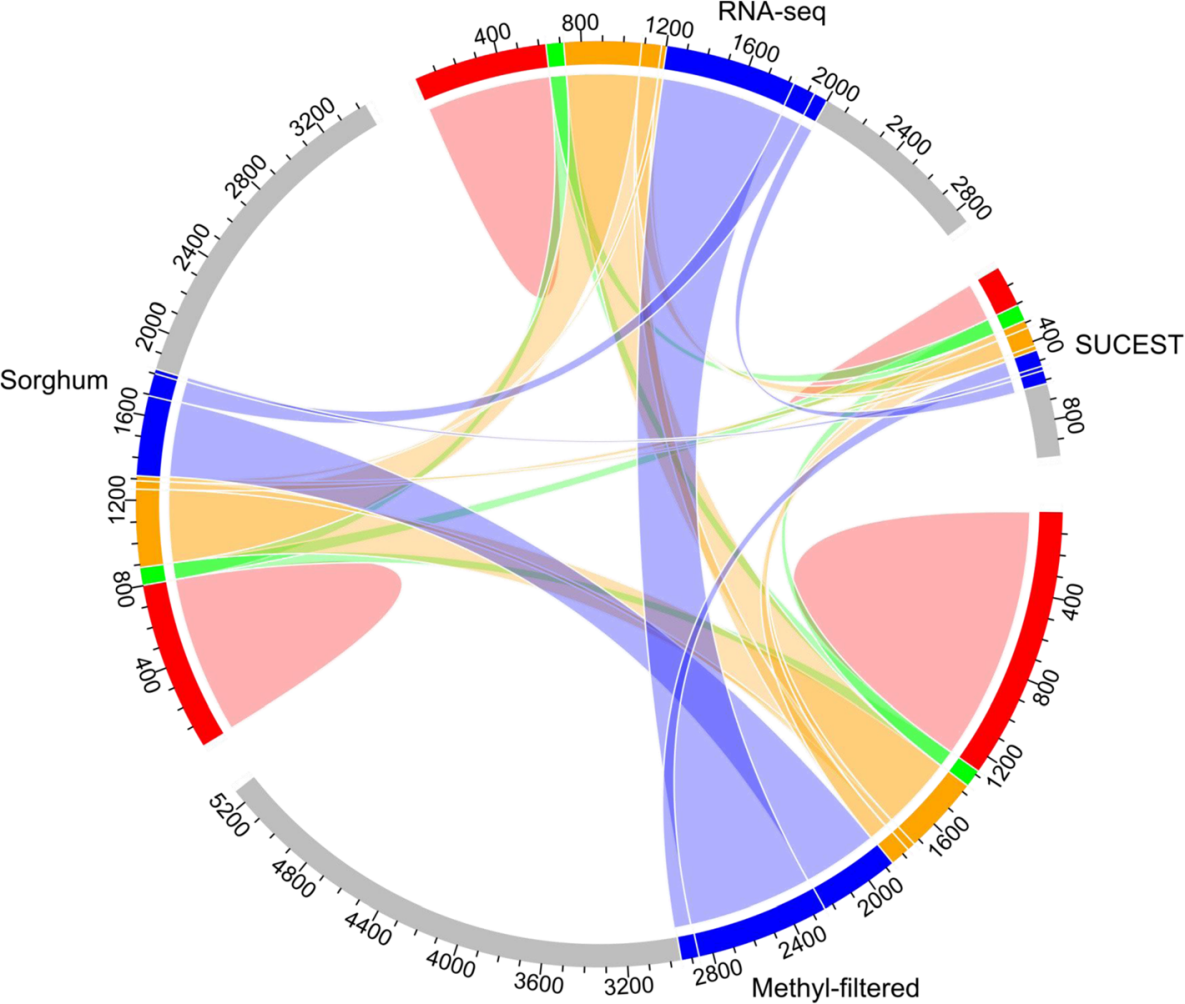
Circular plot showing the redundancy between single-dose markers from four pseudo-references (methyl-filtered sugarcane genome, *Sorghum bicolor* genome, RNA-seq sugarcane transcriptome and SUCEST project sequences) that were used to align the GBS sugarcane tags. The red regions represent redundancy within each pseudo-reference, whereas the green, orange and blue regions represent redundancy between four, three and two pseudo-references, respectively. The remaining grey regions represent loci that are unique to each pseudo-reference

The redundancy of the SDM was inspected after quality and ploidy filtration with the alleles recoded as *a* or *b.* All the references showed very similar levels of redundancy within and between them (Fig. 2). For instance, the same overall level of 22.7% for within-redundancy was found. Only 84 SNPs markers were attributed equally to all four references and represented approximately 3.8% of each reference. Interestingly, each reference provided 39.6% new loci on average. By keeping only one call for each ambiguous marker, we obtained 7,049 loci in total for mapping. Of these loci, 5,757 (81.67%) and 1,292 (18.33%) segregated 1:1 and 1:2:1, respectively (Table 3).

**Table 3:** Overall single-dose gel-based and GBS-based markers screened for the progeny of the cross between sugarcane cultivars SP80-3280 and RB835486

Markers	Gel-based markers			GBS-based markers	
	Genomic SSR	Genic SSR	TRAP		Total
Number of SDMs evaluated (gel-based and GBS-based markers)	109	842	80	7,049	8,080
SDMs with 1:1 segregation	66	456	23	5,757	6,302
SDMs with 1:2:1 segregation (GBS-based markers)	-	-	-	1,292	1,292
Double SDMs (gel-based markers) with 3:1 segregation	43	386	57	-	486
Number of markers with distorted segregation	25	336	41	0	402
Total number (1:1, 1:2:1 and 3:1) feasible for linkage analysis	84	506	39	7,049	7,678

### Gel-based marker genotyping

A total of 120 SSRs and four combinations of TRAP markers (COMT + ARB2, SuPS + ARB1, CCR + ARB1, and CCR + ARB3) produced 1,031 polymorphic bands. Of these 1,031 bands, 545 (52.86%) were tested for 1:1 segregation, and 486 (47.14%) were tested for 3:1 segregation. The number of SDMs available for linkage analysis was 629 (61%), of which 506, 84 and 39 originated from genic SSR, genomic SSR and TRAP markers, respectively (Table 3).

### Genetic map

An integrated genetic map was constructed using 151 full sibs generated from a cross between SP80-3280 and RB835486 (Additional file 1: Figure S1). Of the 8,080 SDMs that were scored (Table 3), 7,678 were used for linkage analysis (7,049 GBS-based markers and 629 gel-based markers), and 993 (12.93%) were placed in the linkage map (Tables 3 and 4). The mapped markers included 934 GBS-based markers and 59 SSRs*.* The distribution of the segregation patterns of mapped markers were 254 B3-type markers (1:2:1), 15 C-type markers (3:1), 518 D1-type markers or that were informative only for SP80-3280 (500 GBS-based markers and 18 gel-based markers) and 206 D2-type markers or that were informative only for RB835486 (180 GBS-based markers and 26 gel-based markers) (Table 4). The markers were distributed throughout the 223 LGs, with a cumulative map length of 3,682.04 cM and an average marker density of 3.70 cM (Table 5). The length of LGs ranged from 1.06 cM (LG 70) to 235.67 cM (LG46), with an average of 16.51 cM; 56 LGs displayed lengths shorter than 2 cM, 95 LGs exhibited lengths greater or equal to 2 cM and smaller than 10 cM, and the other 72 LGs had lengths greater or equal to 10 cM.

**Table 4:** Distribution of the different marker types as mapped according to their cross type

Cross type	Number of markers				
	Gel-based markers				
	Genomic SSR	Genic SSR	TRAP	GBS-based markers	Total
D1.10 (*ab x aa*)	-	-	-	500	500
D1.13 (*ao x oo*)	4	14	0	-	18
D2.15 (*aa x ab*)	-	-	-	180	180
D2.18 (*oo x ao*)	4	22	0	-	26
B3.7 (*ab x ab*)	-	-	-	254	254
C.8 (*ao x ao*)	2	13	0	-	15
Total	10	49	0	993	993

**Table 5:** Number of each type of mapped marker within each homo(eo)logous group (HG), number of linkage groups (LGs) within each HG, the length of each HG in centimorgans (cM) and the marker density in cM of each HG for the genetic map construct from a progeny of a cross between sugarcane cultivars SP80-3280 and RB835486

HG	No. LGs	No. SSR	No. GBS-based markers	No. mapped markers	Length of HG (cM)	Marker density (cM)
1	2	0	11	11	48.90	4.44
2	2	0	8	8	61.72	7.71
3	3	5	21	26	157.14	6.04
4	2	0	9	9	59.09	6.56
5	4	5	17	22	100.45	4.56
6	2	2	14	16	144.24	9.01
7	2	1	9	10	21.45	2.14
8	2	0	8	8	37.84	4.73
9	2	4	5	9	53.72	5.96
10	3	0	19	19	120.08	6.32
11	5	13	31	44	273.66	6.22
12	3	5	7	13	40.64	3.12
13	2	0	7	7	2.91	0.41
14	2	0	9	9	60.21	6.69
15	3	0	13	13	69.55	5.35
16	4	10	8	18	120.73	6.70
17	3	0	14	14	15.35	1.09
18	2	4	3	7	37.26	5.32
Unassigned in HG	175	10	721	730	2,257.10	3.09
Total	223	59	934	993	3,682.04	3.70

A total of 18 HGs were formed based on the common genomic origins of mapped loci from different LGs, which were provided by SSR and GBS-based markers. The number of LGs allocated into HGs ranged from two (HG1, HG2, HG4, HG6, HG7, HG8, HG9, HG13, HG14 and HG18) to five (HG11). The coverage within the HGs varied from 2.91 cM (HG13) to 273.66 cM (HG11). A total of 175 LGs with 730 markers remained unassigned to any HG (Table 5 and Additional file 1: Figure S1).

### QTL mapping

QTL mapping was performed for POL%C, BRIX, SD and FIB traits [10] by applying a CIM model [62] to the integrated genetic map. Considering all traits, 24 cofactors were found for each location, Araras and Ipaussu. The trait with more cofactors was SD, with eight cofactors identified for each location (Additional file 1: Table S2).

To declare the significant QTLs, a permutation test was performed for each phenotype [96]. The values of the *LOD* Score threshold for BRIX, POL%C, SD and FIB at Araras and Ipaussu were 3.79 and 3.77, 3.80 and 3.86, 4.20 and 4.28, and 4.45 and 4.14, respectively. In this case, we were able to declare seven QTLs. For the respective locations, Araras and Ipaussu, BRIX had two and one QTLs, POL%C had one and one QTL, SD had zero and one QTL, and FIB had one and zero QTLs.

**Fig. 3:**
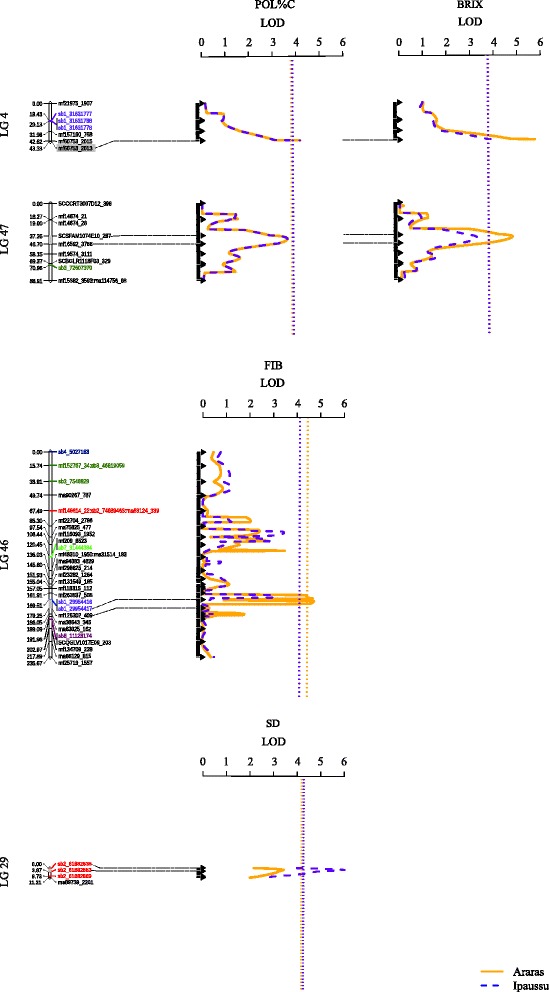
Composite interval mapping (CIM) for soluble solid content (BRIX, in °Brix), sucrose content of cane (POL%C, in %), stalk diameter (SD, in mm) and fiber content (FIB, in %) from the SP80-3280 and RB835486 F1 population. Blue and yellow dotted lines indicate the *LOD* thresholds for Ipaussu-SP and Araras-SP, respectively, obtained after permutation tests. The portions highlighted in gray in the linkage groups show the positions of the QTLs

The global *LOD* Score values ranged from 4.17 to 6.02, and the *R* ^*2*^ values ranged from 2.71% to 9.19%. The highest *LOD* Score for the additive effect was 5.22 for parental SP80-3280 for a QTL associated with BRIX at Araras (B1 at LG4). The highest *LOD* Score for the dominance effect was 3.93 for a QTL associated with FIB at Araras (FIB1 at LG46). The segregation patterns of the QTLs were as follows: 1:1 (42.85%), 1:2:1 (14.30%) and 3:1 (42.85%). The results of the QTL mapping are summarized in Table 6 and Fig. 3.

**Table 6:** QTLs mapped for BRIX, POL%C, SD and FIB traits by applying a CIM model in Araras (Location 1) and Ipaussu (Location 2)

Location	QTL	Trait	LG^a^	Position (cM)^a^	Flanking Markers^b^	Global LOD^b^	R^2 c^	Additive effect SP80-3280^d^	LOD^e^	Additive effect RB835486^d^	LOD^e^	Dominance effect^d^	LOD^e^	Segreg^f^
1	B1	BRIX	4	43.32	mf60753_2013 (QTL)	5.75	9.19	−0.31	5.22	0.08	0.25	0.04	0.09	1:1
1	B2	BRIX	47	40.00	SCSFAM1074E10_287 - QTL - mf16592_3766	4.79	4.78	−0.23	2.22	0.28	3.77	−0.17	1.26	3:1
2	B3	BRIX	4	43.32	mf60753_2013 (QTL)	4.24	7.65	−0.23	3.84	0.06	0.16	0.01	0.01	1:1
1	P1	POL%C	4	43.32	mf60753_2013 (QTL)	4.18	8.10	−0.23	4.14	0.14	0.84	0.08	0.27	1:2:1
2	P2	POL%C	4	43.32	mf60753_2013 (QTL)	4.17	8.09	−0.23	4.13	0.14	0.83	0.08	0.27	1:1
2	SD1	SD	29	2.00	sb2_61882838 - QTL - sb2_61882853	6.02	5.38	0.36	1.66	0.64	4.57	−0.54	3.32	3:1
1	FIB1	FIB	46	171.00	sb1_29954417 - QTL - mf125302_409	4.77	2.71	−0.47	3.11	−0.75	3.95	0.38	3.93	3:1

^a^LG: Linkage groups; Position (cM): QTL position on LG; ^b^Adjacent markers for QTLs and associated LODs; ^c^Explained phenotypic variation; ^d^Additive effects of parents and dominance effects; ^e^LODs of the additive and dominance effects; ^f^Estimation of the segregation pattern of the QTLs

For BRIX, two QTLs (B1 and B2) explained 10.54% of the phenotypic variation in Araras. The QTL identified in Ipaussu (B3 at LG4) was also part of a set found in Araras, *i.e.*, it could be near in the LG at both locations and showed similar effects; this QTL had a significant additive effect for parental SP80-3280 and a segregation pattern of 1:1. In addition, the mapping analysis showed that the region of QTLs B1 and B3 at LG4 was also associated with POL%C (P1 and P2). QTLs for SD and FIB (SD1 and FIB1) showed larger dominance effects that were negative for SD and positive for FIB (Table 6 and Fig. 3).

### Sequence annotation

Sequence similarity was found for six out of seven adjacent markers of the mapped QTLs, with homologies for *S. bicolor*, *Solanum tuberosum* and *Zea mays*. A functional description of the sequences showed possible candidate genes for BRIX, SD and FIB traits, whereas the sequence from the marker associated with the QTL found for POL%C did not show similarity or a characterized protein. Of the total mapped QTLs, three presented adjacent markers and were located in LG47 (B2), LG29 (SD1) and LG46 (FIB1) for BRIX, SD and FIB traits, respectively (Tables 6 and 7).

**Table 7:** Functional description of the sequences that gave rise to adjacent markers of the mapped QTLs for the traits BRIX, POL%C, SD and FIB, and references regarding their functions in plants

Marker	QTLs	LG	Locations	Traits	Description	e-value	Reference
mf60753_2013	B1, B3, P1, P2	4	1 and 2	BRIX and POL%C	No homology found	-	-
SCSFAM1074E10_287	B2	47	1	BRIX	Extended synaptotagmin-1-like [*Zea mays*]	2.6^e-26^	[137–142]
mf16592_3766	B2	47	1	BRIX	Hypothetical protein SORBIDRAFT_03 g038130 [*Sorghum bicolor*]	1.2^e-18^	Unknown function
sb2_61882838	SD1	29	2	SD	Hypothetical protein SORBIDRAFT_02 g026690 [*Sorghum bicolor*]	4.0^e-13^	Unknown function
sb2_61882853	SD1	29	2	SD	Hypothetical protein SORBIDRAFT_02 g026690 [*Sorghum bicolor*]	4.0^e-13^	Unknown function
mf125302_409	FIB1	46	1	FIB	Zinc finger protein CONSTANS-LIKE 15 [*Solanum tuberosum*]	0.0	[143–148]
sb1_29954417	FIB1	46	1	FIB	Transposon mutator sub-class [*Sorghum bicolor*]	2.0^e-177^	[149]

For BRIX, the QTL B2 had two adjacent markers that were each identified in a different reference. The region of the GBS-based marker SCSFAM1074E10_287, which originated from SUCEST sequences, showed similarity with *extended synaptotagmin-1-like*, which is a member of a family of membrane-trafficking proteins. The second adjacent GBS-based marker of this QTL, mf16592_3766, which originated from the methyl-filtered sugarcane genome, showed similarity with a hypothetical protein in *S. bicolor* (Tables 6 and 7).

For SD, the QTL SD1 had two adjacent GBS-based markers, which were both identified from the sorghum genome. The markers sb2_61882838 and sb2_61882853 have a small physical distance in the sorghum genome and share almost the same sequence determine. These two markers showed similarity with a hypothetical protein in *S. bicolor* (Tables 6 and 7).

For FIB, the QTL FIB1 had two adjacent GBS-based markers from two distinct references. Moreover, each of the two adjacent markers showed different similarity by descent. The region of the sb1_29954417, which originated from sequences of the sorghum genome, had similarity with *transposon mutator sub-class*, and the second adjacent marker, mf125302_409, which originated from the methyl-filtered sugarcane genome, had similarity with *zinc finger protein CONSTANS-LIKE 15* (Tables 6 and 7).

## Discussion

The simultaneous identification and genotyping of SNPs and indels is possible because of important recent advances in sequencing [41–50]. GBS is the preferred high-throughput genotyping method for plants with some level of genetic complexity; this method involves complexity reduction and multiplex sequencing to produce high-quality polymorphism data at a relatively low cost per sample [101]. Using four pseudo-references to discover GBS-based markers, we obtained more markers suitable for linkage analysis (Table 3) than any other previously published study on sugarcane mapping. The strategies adopted for the discovery of GBS-based markers allowed us to relate the sugarcane markers to sorghum chromosomes [73] and to potential genetic regions sampled from the methyl-filtered sugarcane genome [72], RNA-seq sugarcane transcriptome [66] and SUCEST project sequences [65].

The highest alignment (87.94%) of the 3.1 million resulting tags against the methyl-filtered sugarcane genome also contains most of the high-quality SDMs (Table 2). This great alignment rate may be related to the greater amount of scaffolds for this reference compared with the other three references and to the fact that the *Pst*I enzyme used for library formation is sensitive to DNA methylation [102]; thus, more polymorphic sites are expected from the methyl-filtered genome. Additionally, GBS-based markers had more markers mapped to parent SP80-3280 than to parent RB835486 (Table 4). This result can be explained by some factors: a) the possible presence of more *Pst*I enzyme restriction sites in cultivar SP80-3280 than in RB835486, leading to more polymorphic sites in the first cultivar, as shown for barley cultivars by Liu et al. [54]; b) also could be due to more similarity between the genome of SP80-3280 with the reference; or c) due to different methylation effects between the two cultivars. Furthermore, inconsistencies in the number of sites sequenced per sample [102] and in the number of reads per site [103, 104], in addition to the filtering steps applied to the GBS libraries to obtain the markers, can influence the observed result. Other factors that can influence these results are the quality and quantity of the biological replicates used for GBS-based marker calling. Because the sequencing of the samples can present failures that will be included in the downstream process, better dosage and ploidy level estimates for each marker in the SuperMASSA software can be hampered.

The analysis of the loci with high coverage that were filtered for missing data after analysis using SuperMASSA software showed that for the ploidy levels under consideration, the number of loci varied within each ploidy class (Fig. 1), suggesting that the number of chromosomes within the HGs is not constant in sugarcane, as reported previously [39, 105]. As stated by Garcia et al. [39], technique artifacts yielding either strong bias or too much noise should explain marker misclassification, *i.e.*, loci not included in the 6-14 expected ploidy range. In fact, the graphical Bayesian model used in the analyses benefits smaller ploidies due to parsimony when the skewed clusters are confounded or, conversely, favors a higher number of clusters by attempting to explain a diffuse scatterplot [106]. In addition, Garcia et al. [39] hypothesized that poor-quality data can also be generated by biological events such as copy number variations or paralogous regions. We used the same *ad hoc* criteria as Garcia et al. [39] to classify each locus into one quality category based on the *a posteriori* probabilities for each ploidy category A as represented by 60.4% of loci of all ploidies on average (Fig. 1), which is smaller than the 77.6% of Sequenom-based data that were previously studied [39]. The GBS read count data worked slightly poorer because of their broad genome coverage and eventual technique artifacts. Despite this reduction, a large part of the loci could be further exploited for mapping purposes.

The presence of repeat elements, paralogs, and incomplete or inaccurate reference genome sequences can create ambiguities in GBS-based marker calling [107]. After we selected the loci that were classified into category A and ploidies ranging from 6 to 14 (Table 2), we continued on to the final steps of quality control and redundancy analyses that showed a low redundancy considering simultaneously all four references. Aitken et al. [35] presented the first sugarcane genetic map with DArT markers and did not remove any redundant markers. Sugarcane has a large and complex genome, and a low level of redundancy is important for showing the true coverage of the genome. Heslot et al. [52] showed that DArT markers were significantly more redundant than GBS markers, and they suggested that GBS markers were significantly more evenly distributed across the wheat genome. These authors also concluded that GBS is the marker platform of choice for further diversity analyses and genomic selection.

The integrated genetic map of sugarcane obtained in this paper presents improvements in comparison with previous works. Here, the number of markers used for linkage analysis is more than twice the number of markers used for the development of the largest map [35]. Furthermore, this genetic map of sugarcane is the first to use a high-throughput approach for genotyping. Co-dominant biallelic markers can segregate in a 1:2:1 fashion (‘B3’ cross type), which is even more informative for map integration purposes. The previously published genetic maps had molecular markers treated as dominant even when are potentially co-dominant, that is, all clusters that have at least one copy of the allele will collapse into a single cluster [37]. The mapping population was formed by a cross between polyploid heterozygous parents, and for each segregating loci, there could be different numbers of segregating alleles and different dosages that are potentially expressed. Thus, accessing the dosage information of the SDMs with a segregation pattern of 1:2:1 was important; the double SDMs with a segregation pattern of 3:1 (‘C8’ type) were also important. This information was used to construct an integrated genetic map for sugarcane that increased the genome coverage.

The 223 LGs obtained here had a cumulative map length of 3,682.04 cM and an average marker density of 3.70. The number of LGs exceeds the number of chromosomes of modern sugarcane cultivars, which can range from 100 to 130 [1, 9], and 56 LGs showed lengths shorter than 2 cM. This result indicates the presence of gaps and that the map is not yet well saturated. In 2007, Oliveira and collaborators [38] claimed that because there is a constraint to discarding markers in multiples doses, *i.e.*, duplexes of monoparental origin, triplex or higher multiplex markers, gaps are evidently expected; the same discussion should be applied to this study. The number of unlinked markers (87.07%) is higher than that obtained in other sugarcane maps [4, 31, 35, 37, 38, 81, 108–110] and reflects the highly stringent criteria used to construct an integrated genetic map of sugarcane that is reliable for performing QTL mapping analysis.

To increase the understanding of the genetic architecture of sugarcane, a necessary requirement is the availability of good genetics maps, *i.e.*, maps with a high density of markers and with high coverage of the genome [111–113]. The complexity of the sugarcane genome, the cost of generating a large number of markers, and the absence of a statistical genetic model that could consider other segregation ratios beyond 1:1, 1:2:1 and 3:1 have limited the development of high-density genetic maps. These limitations have delayed practical applications of genomic tools in sugarcane, in contrast to other crops that have already advanced to MAS and genomic selection. Sugarcane still does not have its genome completely sequenced, and sorghum genome is widely recognized as a reference genome for comparative analysis with sugarcane [67]. The origin of modern sugarcane cultivars raises issues that are related to not only the extent and nature of the divergence of the sugarcane and sorghum genomes but also the relations (in terms of meiosis and dosage) among homo(eo)logous loci [68]. Differences in chromosome structures between the ancestor species and pairing behavior in modern cultivars suggest that the hybrid monoploid number is likely to be greater than 10 in sugarcane hybrids [38, 114]. Probably because of aneuploidy, an unequal number of chromosomes in each HG is likely to occur; this inequality was reflected in the 18 HGs with differences in genome coverage. Moreover, translocation events may have occurred in sugarcane between regions equivalent to sorghum, as discussed previously [27, 28, 115–118]. Although these comparative studies proposed hypotheses about the evolutionary aspects of sugarcane and sorghum, the results showed variations that were primarily derived from the low resolution of the genetic maps used and to the coverage of the sugarcane genome. In addition, it is important to highlight that advances in the assembly of polyploid genomes will enable the use of the full sugarcane genome as a reference in the future [119].

The genetic maps and field data obtained through designed experiments are required for QTL mapping studies. For sugarcane, multiple harvest-location trials may be used to infer the genetic architecture of quantitative traits. However, this inference makes the data analysis more complex and challenging because of the interactions that it generates, *e.g.*, genotype by environment interactions. To solve this problem, a mixed model approach has been used to obtain highly accurate genetic estimates [10, 31, 120], and for segregating populations, these results will be the input for QTL mapping.

In this study, QTL mapping was performed by applying the statistical model proposed by Gazaffi et al. [62], which extends the CIM [64] for a full-sib progeny. The primary advantage of CIM is that it is more precise and effective at mapping QTLs in comparison with single-marker analysis and IM, especially when QTLs are present outside the mapping window [64]. The results obtained from the CIM method are usually comparable to those obtained from multi-QTL analysis if a high-density genetic map is employed to better represent the number of loci underlying the quantitative traits [79]. Several QTL mapping studies in sugarcane have been published [31, 34, 61, 81, 121–136]. The comparison between the results may be biased by several issues, such as the different rates of polymorphisms in parents, the number of progeny, the evaluation methodologies for phenotypic traits, the methodologies used for QTL detection, genetic map contruction, and experimental design, among others. For example, Pastina et al. [31] worked with a population of 100 individuals from a cross between cultivars SP80-180 and SP80-4966 to construct an integrated genetic map that was 2,468.14 cM in length. These researchers used IM to test presence of putative QTLs and a multi-QTL model to declare QTLs and found 46 QTLs. There were 13 mapped QTLs for the tonnes of cane per hectare (TCH), 14 for sugar content in tonnes of sucrose per hectare (TSH), 11 for FIB and eight for POL%C. Singh et al. [34] studied the progeny of 207 individuals derived from a cross between cultivars Co86011 and CoH70, and they constructed two separate genetic maps, one for each parent. Through the CIM model, these researchers found 31 QTLs, with seven for BRIX and four for stalk number (SN). Thus, specific objectives must be taken into consideration for QTL mapping in sugarcane.

For QTL mapping in this study, we performed a permutation test to obtain the threshold for declaring significant QTLs [97]. The CIM model was a useful tool once it was able to identify regions with next QTL considering Araras and Ipaussu over the harvests (three years of evaluations). Seven QTLs were identified, being that a region located in LG4 at 43.32 cM showed QTLs for BRIX (B1-B3) and POL%C (P1-P2). The marker associated with the QTLs was identical for both traits, and the region that gave rise to this marker could be evaluated for future applications by sugarcane breeding programs. POL%C and BRIX are correlated traits [10], and although the commercial cultivars used as parents of the mapping population presented a small contrast in terms of phenotypic averages, especially for sucrose content, these results show that a combination of different alleles in each parent segregates and contributes to the observed variation in progeny. Furthermore, this result was expected because the parents of the mapping population are cultivars that were improved primarily to increase the sucrose content.

The percentage of phenotypic variation explained by each QTL ranged from 2.71% (FIB1) to 9.19% (B1) for Araras and from 5.38% (SD1) to 8.09% (P1) for Ipaussu. The sampling of the genome in single doses requires that QTL also segregate as single doses. Furthermore, the use of improved parents that have close phenotypic averages and that have some level of fixed alleles for traits of interest could decrease the chances of detecting QTLs with high rates of explained phenotypic variation. Nevertheless, the QTLs described here can be considered reliable because they have all taken into account the phenotypic average of three harvests and because it was identified QTLs in same position into LG4 for both locations.

The common QTLs between locations may be regarded as potential regions to search genes that are involved in controlling quantitative traits. In addition, a strong marker-QTL association detected in full-sib progenies also could be an impact on crop improvement via clonal propagation because probability of crossover between the marker and the QTL is low [34]. Therefore, the similarity analysis and annotation of sequences that originated the markers with mapped QTLs are important for identified putative candidate genes in sugarcane, although the QTLs regions are relatively large and an uncertain number of genes may be involved with the evaluated traits. Sugarcane has high genetic complexity and its genome still does not was completely sequenced [67], whereas inferences about putative candidate genes could be contribute for new insights and open new fronts of research to mining and validation of genes of interest.

For the BRIX trait, we can highlight the similarity of the marker SCSFAM1074E10_287, located in QTL B2, with *extended synaptotagmin-1-like*, which is a member of a membrane-trafficking protein family that is characterized by an N-terminal transmembrane region, a linker of variable size, and two C-terminal C2 domains in tandem [137]. C2 domains, identified as a conserved sequence motif in protein kinase C [138], are autonomously folded protein modules that generally act as calcium (Ca^2+^) and phospholipid-binding domains and that were shown to represent autonomously folded Ca^2+^-binding domains in synaptotagmins [139]. In addition, Ca^2+^ acts as a second messenger in the signal transduction pathways of hormones and environmental stimuli (touch, wind, chilling, light, and elicitors) [140], and several proteins that are involved in photosynthesis depend on Ca^2+^ [141]. In sugarcane, Papini-Terzi et al. [142] identified differentially expressed genes in genotypes contrasting for sucrose content and showed that among these genes, nine were associated with calcium signaling and a calcium-dependent protein kinase (ScCDPK-27). Further researches are needed to expand the inference found in this study for pathways and regulatory networks of sugarcane in order to relate with sucrose biosynthesis and, consequentely, with BRIX trait.

For the FIB trait, a QTL (FIB1) showed different similarities for each of the two adjacent markers and we can highlight the similarity of the marker mf125302_409 with *zinc finger protein CONSTANS-LIKE 15*. The CONSTANS (CO) protein is a zinc finger transcription factor that contain two conserved domains (*i.e.*, a B-box zinc finger domain and a CCT [CO, CO-like, TOC1] domain), located in the region near the amino- and carboxy-terminus, respectively [143, 144]. The CO proteins play a central role in the photoperiod pathway of *Arabidopsis* by mediating the circadian clock and floral integrators via positive regulation of FLOWERING LOCUS T expression [145–147]. In cotton (*Gossypium* spp.), which is the most important natural source of fiber for the textile industry, the CO5 protein (*CONSTANS-LIKE 5*) was up-regulated for cell wall modification and developing fibers in MD52ne, a near-isogenic line. Furthermore, using an F_2_ population derived from a cross between MD52ne and MD90ne, stable QTLs for bundle fiber strength and fiber length were found, and CO5 was present on the QTL region for fiber length [148]. Although there is an indicative of relation between the candidate gene and FIB trait, advanced studies should be conducted to validate and prove the real function and effects into phenotypic expression in sugarcane.

## Conclusions

Our understanding of the genetic architecture of triats of interest in sugarcane is increasing with the development of new analytical methods. The estimation of ploidy and allelic dosage through markers generated by GBS as well as the inclusion of these markers in an integrated genetic map of sugarcane were first observed in this study, and these markers showed great potential for QTL mapping. The CIM approach that provided additive and dominance effects and estimated the segregation patterns for all mapped QTLs was efficient for detecting possible stable QTLs among the evaluated locations. The verification of possible candidate genes for mapped QTLs as a preliminary analysis showed importance for new insights into the comprehensive relations between phenotypes and genotypes. It is still necessary to develop statistical approaches to enable the inclusion of markers at multiple doses to enhance the coverage by linking the SDMs that are pulverized by the genome. Moreover, QTL mapping with markers in multiple doses must be considered a major step in the understanding of regions that control quantitative traits in polyploid organisms and perhaps permit the verification of the allelic expression of phenotypic traits in the future.

## Additional file

Additional file 1: Table S1.
Bowtie2 alignment results of 3,103,708 million GBS tags in absolute and relative (in parentheses) values. **Table S2.** Markers selected as cofactors for mapping through the composite interval mapping (CIM) model for the soluble solid content (BRIX, in °Brix), sucrose content of cane (POL%C, in %), fiber content (FIB, in %) and stalk diameter (SD, in mm) traits for the locations Araras-SP and Ipaussu-SP. **Figure S1.** Genetic map of sugarcane that was generated with a population of 151 full sibs that originated from a commercial cross between the cultivars SP80-3280 and RB835486. The linkage groups (LGs) were separated into 18 homo(eo)logous groups (HGs). The numbers on the left of the LGs are the cumulative genetic distances in Kosambi centimorgans. The marker names are shown on the right. Markers corresponding to each sorghum chromosome are highlighted in different colors. (DOCX 2545 kb)

## Abbreviations

BRIX: Soluble solid content
CCR: *Cinnamoyl-CoA reductase*
CIM: Composite interval mapping
COMT: *Caffeic acid 3-O-methyltransferase*
DNA: Deoxyribonucleic acid
FIB: Fiber content
GBS: Genotyping-by-sequencing
HGs: Homo(eo)logous groups
IM: Interval mapping
LGs: Linkage groups
LOD: Logarithm of the odds
MAP: Maximum *a posteriori*
MAS: Marker-assisted selection
MDM: Multiple dose marker
NGS: Next-generation sequencing
PCR: Polymerase chain reaction
POL%C: Sucrose content of cane
QTLs: Quantitative trait loci
RNA: Ribonucleic acid
SD: Stalk diameter
SDMs: Single-dose markers
SNPs: Single nucleotide polymorphisms
SSRs: Single sequence repeats, another term for microsatellites
SuPS: *Sucrose phosphate synthase*
TRAP: Target region amplification polymorphism
VCF: Variant call format
