# Supplementary material for: GBS-based single dosage markers for linkage and QTL mapping allow gene mining for yield-related traits in sugarcane
Source: BMC Genomics. 2017 Jan 11;18:72. doi: 10.1186/s12864-016-3383-x (PMC5225503; doi:10.1186/s12864-016-3383-x)
Supplement: Additional file 1: Table S1. — Bowtie2 alignment results of 3,103,708 million GBS tags in absolute and relative (in parentheses) values. Table S2. Markers selected as cofactors for mapping through the composite interval mapping (CIM) model for the soluble solid content (BRIX, in °Brix), sucrose content of cane (POL%C, in %), fiber content (FIB, in %) and stalk diameter (SD, in mm) traits for the locations Araras-SP and Ipaussu-SP. Figure S1. Genetic map of sugarcane that was generated with a population of 151 full sibs that originated from a commercial cross between the cultivars SP80-3280 and RB835486. The linkage groups (LGs) were separated into 18 homo(eo)logous groups (HGs). The numbers on the left of the LGs are the cumulative genetic distances in Kosambi centimorgans. The marker names are shown on the right. Markers corresponding to each sorghum chromosome are highlighted in different colors. (DOCX 2545 kb) [file 12864_2016_3383_MOESM1_ESM.docx]

**Additional file 1**

**Table S1**. Bowtie2 alignment results of 3,103,708 million GBS tags in absolute and relative (in parentheses) values.

| GBS-Tassel pipeline pseudo-references | Non-aligned tags | Aligned tags | | |
| --- | --- | --- | --- | --- |
|  |  | Overall alignment | Unique alignment | Non-unique alignment |
| Methyl-filtered sugarcane genome | 374,251  (12.06%) | 2,729,457 (87.94%) | 1,823,623 (58.76%) | 905,834  (29.18%) |
| *Sorghum bicolor* genome (v. 2.1) | 1,791,047 (57.71%) | 1,312,661 (42.29%) | 1,060,705 (34.17%) | 251,956 0  (8.12%) |
| RNA-seq sugarcane transcriptome | 1,907,985 (61.47%) | 1,195,723 (38.53%) | 1,099,697 (35.43%) | 96,026 0  (3.10%) |
| SUCEST project sequences | 2,362,171 (76.11%) | 741,537  (23.89%) | 256,450 0  (8.26%) | 485,087  (15.63%) |

**Table S2**. Markers selected as cofactors for mapping through the composite interval mapping (CIM) model for the soluble solid content (BRIX, in °Brix), sucrose content of cane (POL%C, in %), fiber content (FIB, in %) and stalk diameter (SD, in mm) traits for the locations Araras-SP and Ipaussu-SP.

| Location | Trait | LG | Marker selected as cofactors  for the CIM model | Location | Trait | LG | Marker selected as cofactors  for the CIM model |
| --- | --- | --- | --- | --- | --- | --- | --- |
| Araras | BRIX | 4 | mf60753_2013 | Araras | FIB | 122 | sb9_2129715 |
| Araras | BRIX | 6 | mf62649_246 | Araras | FIB | 124 | mf333_8772 |
| Araras | BRIX | 26 | sb8_1755137 | Araras | FIB | 167 | mf173642_165 |
| Araras | BRIX | 42 | mf229549_282 | Ipaussu | FIB | 47 | sb3_72607370 |
| Araras | BRIX | 47 | SCSFAM1074E10_287 | Ipaussu | FIB | 54 | sb3_63080136 |
| Ipaussu | BRIX | 4 | mf60753_2013 | Ipaussu | FIB | 60 | mf39868_1370 |
| Ipaussu | BRIX | 6 | mf62649_246 | Ipaussu | FIB | 124 | mf333_8772 |
| Ipaussu | BRIX | 26 | sb8_1755137 | Ipaussu | FIB | 167 | mf173642_165 |
| Ipaussu | BRIX | 42 | mf229549_282 | Araras | SD | 7 | sb1_60124665 |
| Ipaussu | BRIX | 46 | mf209_8523 | Araras | SD | 35 | SCJFFL1C04F08_496 |
| Ipaussu | BRIX | 98 | mf17470_1855:rna71385_1041 | Araras | SD | 106 | rna104430_132 |
| Araras | POL%C | 4 | mf60753_2013 | Araras | SD | 133 | rna74863_164 |
| Araras | POL%C | 6 | mf62649_246 | Araras | SD | 146 | sb10_12120909 |
| Araras | POL%C | 26 | sb8_1755137 | Araras | SD | 150 | rna90367_978 |
| Araras | POL%C | 42 | mf229549_282 | Araras | SD | 174 | mf56117_119 |
| Araras | POL%C | 51 | sb3_72553815 | Araras | SD | 218 | rna42720_590 |
| Ipaussu | POL%C | 4 | mf60753_2013 | Ipaussu | SD | 7 | sb1_60124665 |
| Ipaussu | POL%C | 6 | mf62649_246 | Ipaussu | SD | 13 | sb1_3852034 |
| Ipaussu | POL%C | 26 | sb8_1755137 | Ipaussu | SD | 35 | SCJFFL1C04F08_496 |
| Ipaussu | POL%C | 42 | mf229549_282 | Ipaussu | SD | 96 | rna73797_179 |
| Ipaussu | POL%C | 51 | sb3_72553815 | Ipaussu | SD | 106 | rna104430_132 |
| Araras | FIB | 46 | rna90267_787 | Ipaussu | SD | 133 | rna74863_164 |
| Araras | FIB | 47 | sb3_72607370 | Ipaussu | SD | 150 | rna90367_978 |
| Araras | FIB | 60 | mf39868_1370 | Ipaussu | SD | 174 | rna43594_177 |

**
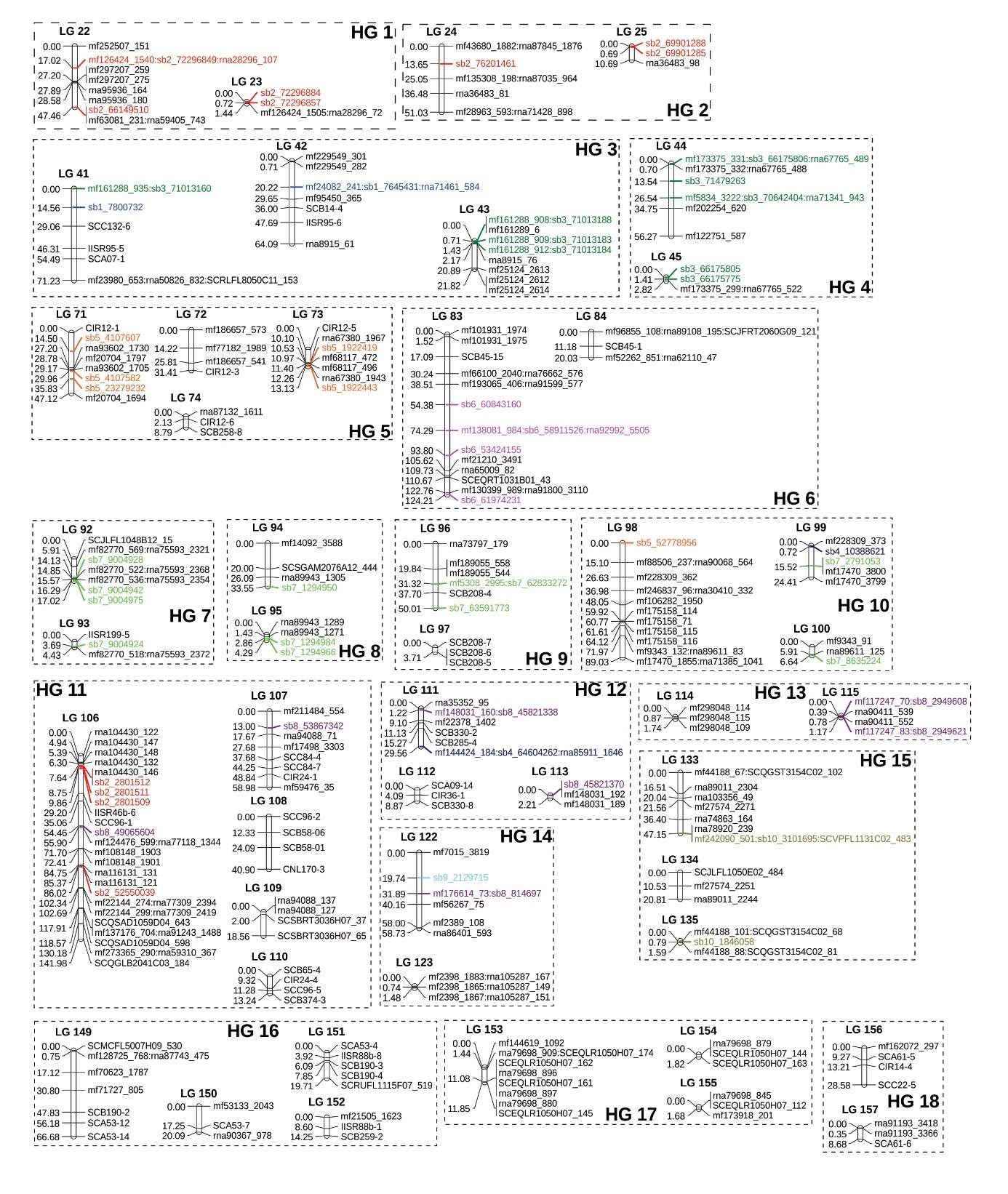
**

**Figure S1**. Genetic map of sugarcane that was generated with a population of 151 full sibs that originated from a commercial cross between the cultivars SP80-3280 and RB835486. The linkage groups (LGs) were separated into 18 homo(eo)logous groups (HGs). The numbers on the left of the LGs are the cumulative genetic distances in Kosambi centimorgans. The marker names are shown on the right. Markers corresponding to each sorghum chromosome are highlighted in different colors.


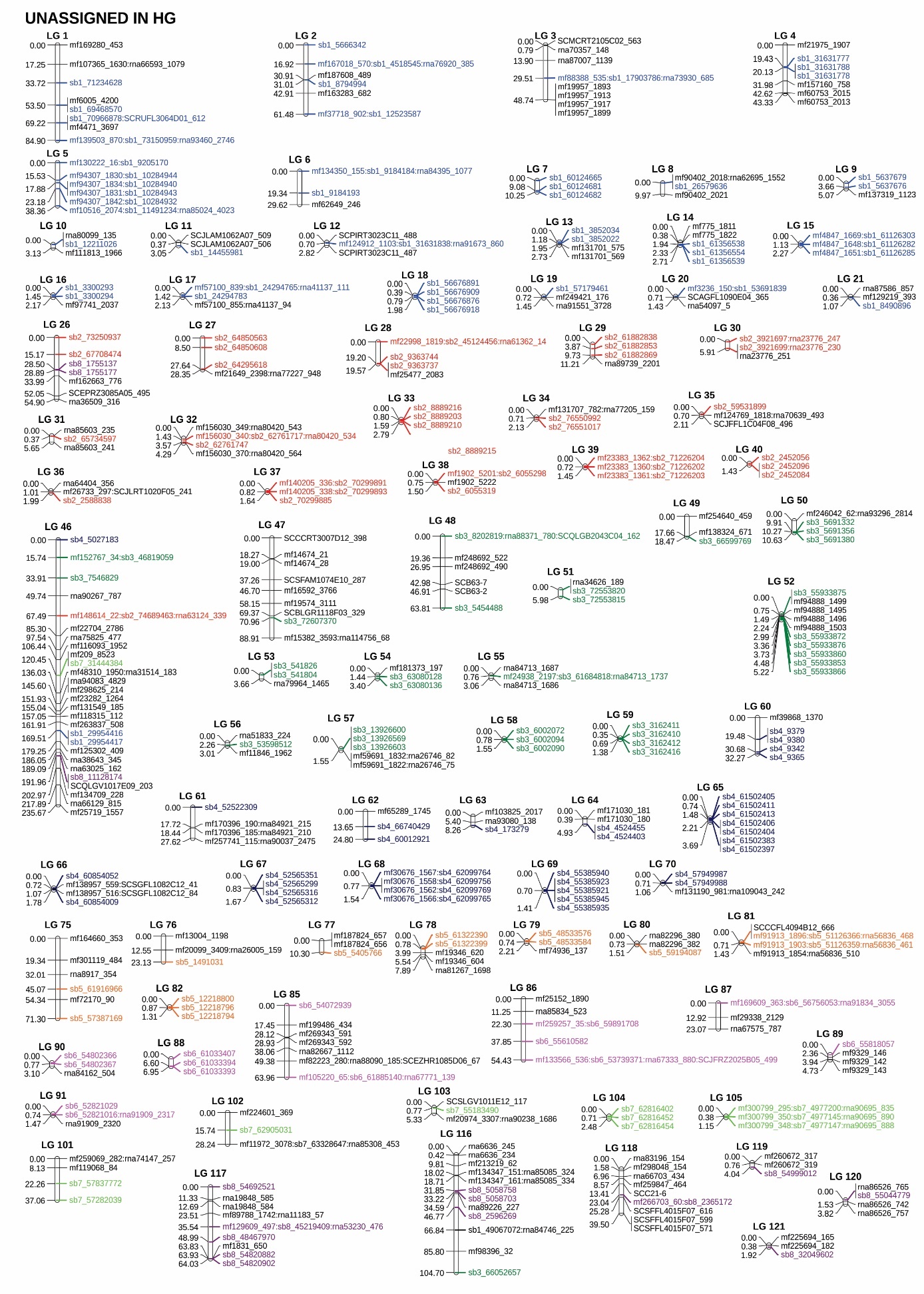


**Figure S1**. Continued.

**
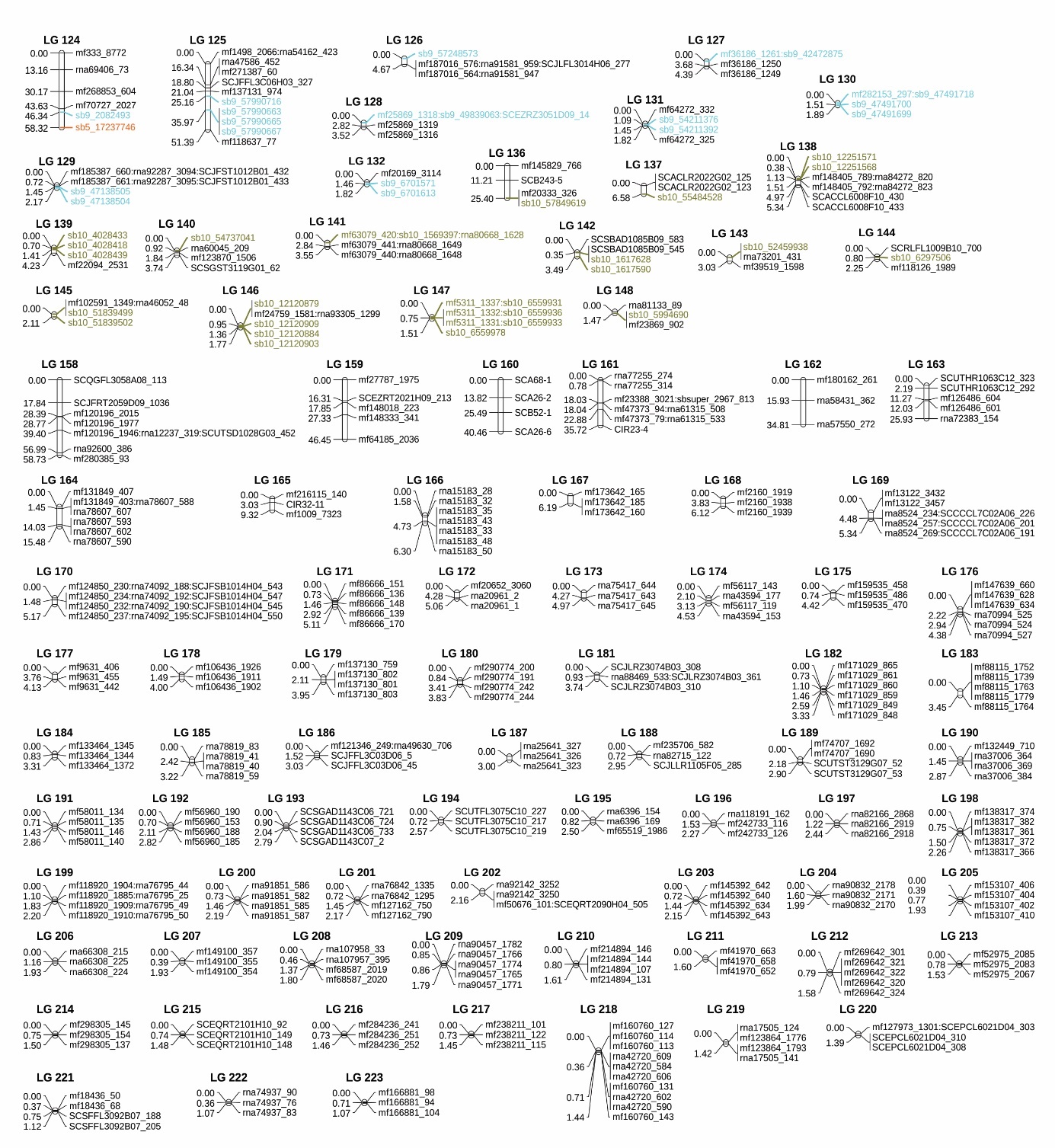
**

**Figure S1**. Continued.
